# SARS-CoV-2 associated rhabdomyolysis in 32 patients

**DOI:** 10.3906/sag-2012-327

**Published:** 2021-06-28

**Authors:** JOSEF FINSTERER, FULVIO SCORZA

**Affiliations:** 1 Department of Neurology, Klinik Landstrasse, Messerli Institute, Vienna Austria; 2 Department of Neuroscience. Federal University of São Paulo/Escola Paulista de Medicina (UNIFESP/EPM), São Paulo Brazil

**Keywords:** Rhabdomyolysis, coronavirus, ground glass opacities, skeletal muscle, myocyte necrosis

## Abstract

**Background/aim:**

This mini-review aims at summarising and discussing previous and recent findings concerning the clinical manifestations, therapeutic management, and outcome of SARS-CoV-2 associated rhabdomyolysis.

**Materials and methods:**

Literature search in the PubMed database by applying appropriate search terms.

**Results:**

A total of 26 articles reporting SARS-CoV-2 associated rhabdomyolysis in 32 patients were identified. Age ranged from 16 to 80 years. Four patients were female and 25 were male. Onset of rhabdomyolysis was prior to onset of COVID-19 in 7 patients, and after onset of COVID-19 in the remaining patients. Exposure to myotoxic medication was identified in 18 patients. Myotoxic drugs these patients were taking at the time rhabdomyolysis included azithromycin, hydroxy-chloroquine, placitaxel, propofol, imastinib, piperacillin and meropenem, hydrochlorothiazide, and acetaminophen. Peak creatine-kinase values ranged from 328 to >427656 U/l. The outcome was unreported in 8 cases, favourable in 15 partial, incomplete in 3 cases, and lethal in 6 cases.

**Conclusion:**

SARS-CoV-2 associated rhabdomyolysis is rare, may be most frequently due to the side effects of myotoxic anti-COVID-19 drugs, and only rarely due to virus myositis, and may have a favourable outcome in most patients.

## 1. Introduction 

Since the outbreak of the SARS-CoV-2 pandemic in December 2019 it became increasingly evident that not only the lungs (COVID-19) but also other organs and tissues, including the skeletal muscle, may be primarily or secondarily involved in the infection [1]. Muscle involvement in COVID-19 includes myositis, myalgia, weakness of limb, bulbar, and extra-ocular muscles, exercise intolerance, critical ill myopathy, and rhabdomyolysis [2]. Rhabdomyolysis may be mild, moderate, or severe, may be the initial manifestation of the infection [3], may be complicated by acute renal insufficiency requiring hemodialysis [4], and may have a favourable or fatal outcome (Table). This mini-review aims at summarising and discussing previous and recent findings concerning the clinical manifestations, therapeutic management, and outcome of SARS-CoV-2 associated rhabdomyolysis. 

**Table:** Patients with rhabdomyolysis attributed to an infection with SARS-CoV-2.

Age/NOP Sex ORM EMD CP PCK TR OC REF

15 m B no myalgia, vomiting 21876 FL AL recovery [5]
48 m A yes nr 10768 AV nr [6]
46 f A paclitaxel myalgia, vomiting 87456 AL, ST recovery [7]
38 m A propofol dark urine 21000* AV, HD recovery [8]
Nr nr B no myalgia# 25384 FL ICU [9]
35 f B no myalgia, diarrhoea 71000 FL nr [10]
37 n A yes nr 35000 FL, HD, AV death [Chong, 2020]
46 m A imastinib myalgia >400000 FL, AL death [Solis, 2020]
27 f A piperacillin nr 13732* HF, AV recovery [Kolkova 2020]
meropenem
16 m A HCT myalgia, dark urine >426700 FL, DR, HD recovery [Samies 2019]
49 m A questionable myalgia 23800 FL, DR recovery [Mukherjee 2020]
39 m A colchizine myalgia 17070 nr recovery [Legrand 2020]
71 m A acetaminophen myalgia, fever 8720 FL, AL recovery [Valente-Acosta 2020]
78 m A drugs nr myalgia 22511 FL recovery [Rivas-Garcia 2020]
60 m B no myalgia, fatigue 4287 FL, AL recovery [Borku Uysal 2020]
38 m A no myalgia 42670 FL recovery [Zhang 2020]
16 m A acetaminophen myalgia 427656 FL, AL recovery [Gefen 2020]
80 m B statins, furosemide myalgia, weakness 13581 FL, DR recovery [Suwanwongse 2020]
Nr m A neuroleptics nr 120000 dantrolen$ ICU [Kajani 2020}
75 f B drugs nr none 2767 FL recovery [Chan 2020]
71 m B drugs nr leg twitching 1859 HD, AF ICU [Chan 2020]
16 m B no myalgia 392488 ST nr [3]
67 m A yes none 19773 HD, AV death [4]
39 m A yes macrohematurea 4330 none death [4]
43 m A yes nr 9793 none death [4]
70 m A steroids macrohematurea 5008 FL, ST, AB death [4]
51 m nr nr nr 328 nr nr [Su 2020]
66 m nr nr nr 1001 nr nr [Su 2020]
70 m nr nr nr 2459 nr nr [Su 2020]
69 m A yes myalgia, FT 17434 FL, AL, IG recovery [Jin 2020]
Nr nr A nr nr nr nr nr [Guan 2020]
Nr nr A nr nr nr nr nr [Guan 2020]

A: after onset of COVID-19 or treatment, AL: alkalisation, AV. artificial ventilation, B. before onset of COVID-19 manifestations and treatment, CP: clinical presentation in addition to COVID-19, DR: diuretics, EMD: exposure to myotoxic drugs, FL: fluids, FT: fatigue, HCT: hydrochlorothiazide, HD: hemodialysis, IG: immunoglobulins, nr: not reported, OC: outcome, ORM: onset of rhabdomyolysis, PCK: peak creatine-kinase in U/L, ST: steroids, TR: treatment of rhabdomyolysis, *: myoglobin in mg/L, #: MRI-confirmed myositis, $: malignant neuroleptic syndrome was diagnosed.

## 2. Methods and materials

A literature search in the PubMed database applying search terms “rhabdomyolysis”, “muscle cell necrosis”, “myoglobinuria”, and “creatine-kinase” in combination with “SARS-CoV-2”, “COVID-19”, and “coronavirus” was conducted. Additionally, reference lists were checked for further articles meeting the search criteria. Excluded were articles in languages other than English, French, Spanish, Italian, or German.

## 3. Results

We identified 26 articles reporting SARS-CoV-2 associated rhabdomyolysis in 32 patients (Table). Age, reported in 28 cases, ranged from 16 to 80 years (Table). Sex, reported in 29 patients, was female in 4 and male in 25 patients. Onset of rhabdomyolysis, reported in 29 cases, was prior to onset of COVID-19 in seven patients, and after onset of COVID-19 in the remaining patients. Exposure to myotoxic medication could be identified in 18 patients. Myotoxic drugs these patients were taking at the time rhabdomyolysis occurred included azithromycin, hydroxy-chloroquine, placitaxel [5], propofol [6], imastinib, piperacillin and meropenem, hydrochlorothiazide, and acetaminophen (Table). Typical clinical manifestations of rhabdomyolysis (myalgia, dark urine, fever, fatigue, vomiting, diarrhoea) were reported in 20 patients and remained unreported in 10 cases. Rhabdomyolysis remained asymptomatic in 2 cases. Peak creatine-kinase values ranged between 328 and >427656 U/l (Table). Treatment of rhabdomyolysis was reported in 26 cases included fluids (n = 16), diuretics (n = 3), alkalisation (n = 7), steroids (n = 3), antibiotics (n = 1, and hemodialysis (n = 4) (Table). The outcome was unreported in 8 cases, favourable in 15 cases, incomplete in 3 cases, and lethal in 6 cases (Table). 

## 4. Discussion

Rhabdomyolysis is an acute condition due to damage of myocytes in a single muscle, a group of muscles, or all striated muscles. The three cardinal manifestations of rhabdomyolysis are black tea coloured (dark) urine, myalgia, and fever. More rare clinical manifestations are muscle weakness, vomiting, and confusion. Rhabdomyolysis is diagnosed upon the clinical presentation and laboratory tests, showing marked elevation of creatine-kinase, transaminases, or myoglobin, electrolyte disturbances, and renal insufficiency. Importantly, after resolution of rhabdomyolysis, patients should undergo a neurological exam, needle electromyography, and eventually muscle biopsy or genetic tests. Rhabdomyolysis may result from direct myocyte injury or failure of energy production, leading to an unregulated increase in intracellular calcium and cellular lysis. Accordingly, there are multiple causes of rhabdomyolysis but the most frequent include crush injury, strenuous exercise, medication, drug abuse, infections, and sepsis. More rarely, rhabdomyolysis is due to endocrine abnormalities, electrical injury, heat stroke, prolonged immobilisation, arterial occlusion, snake bites, or inherited muscle disease. There are indications that rhabdomyolysis secondary to an infectious aetiology may be due to direct damage by the pathogen or due to an exaggerated inflammatory response. Similar theories have been proposed for SARS-CoV-2-associated rhabdomyolysis [7]. Viruses which may potentially cause rhabdomyolysis include influenza, HIV, enteroviruses, Epstein-Barr virus, cytomegalovirus, adenovirus, herpes simplex, varicella virus, Zika, Dengue, Coxsackie-B, Herpes-6, Chikungunya, arboviruses, parainfluenza, and metapneumovirus.

Among the eight patients in whom rhabdomyolysis occurred prior to clinical manifestations of COVID-19, no exposure to myotoxic drugs has been identified in seven patients (Table). Whether SARS-CoV-2 in these seven patients was truly the trigger of rhabdomyolysis remains speculative. Subclinical hereditary myopathy has not been excluded in any of them. The case reported by Beydon et al. is the only one in which rhabdomyolysis was due to myositis as confirmed by muscle MRI of lower legs [8]. In the three patients reported by Su et al. creatine-kinase increase was only mild why the diagnosis rhabdomyolysis is questionable. Overall, the frequency of rhabdomyolysis in COVID-19 patients is lower than in patients with MERS-CoV of whom 14.4% experienced rhabdomyolysis. 

In conclusion, SARS-CoV-2 associated rhabdomyolysis is rare, may be most frequently due to side effects of myotoxic compounds given to treat the infection and only rarely due to virus myositis, and may have a favourable outcome in most patients. COVID-19 patients should not receive myotoxic compounds. COVID-19 associated rhabdomyolysis requires further work-up for differentials of rhabdomyolysis after recovery. Not to miss muscle damage associated with COVID-19 high clinical suspicion must be held for any patient with COVID-19 demonstrating signs or symptoms of rhabdomyolysis. 

## Funding

No funding was received.

## Author contribution

JF: design, literature search, discussion, first draft, critical comments.

## Informed consent

Informed consent was obtained.

## Ethical approval

The study was approved by the institutional review board (board decision number?)
